# Integrated bioinformatics analysis of dendritic cells hub genes reveal potential early tuberculosis diagnostic markers

**DOI:** 10.1186/s12920-023-01646-0

**Published:** 2023-09-08

**Authors:** Xiao Wu, Kewei Liu, Shanshan Li, Weicong Ren, Wei Wang, Yuanyuan Shang, Fuzhen Zhang, Yingying Huang, Yu Pang, Mengqiu Gao

**Affiliations:** 1 Department of Bacteriology and Immunology, Beijing Chest Hospital, Capital Medical University, Beijing Tuberculosis & Thoracic Tumor Research Institute, Beijing, 101149 P. R. China; 2 Department of Tuberculosis, Beijing Chest Hospital, Capital Medical University/Beijing Tuberculosis & Thoracic Tumor Research Institute, Beijing, 101149 P. R. China; 3 Jining Medical University, Shandong, 272002 China; 4 Qingdao Mental Health Center, Shandong, 266034 China

**Keywords:** Tuberculosis, Dendritic cell, Hub gene, Bioinformatics, IFN-γ response

## Abstract

**Background:**

Dendritic cells (DCs) are most potent antigen-processing cells and play key roles in host defense against *Mycobacterium tuberculosis* (MTB) infection. In this study, hub genes in DCs during MTB infection were first investigated using bioinformatics approaches and further validated in Monocyte-derived DCs.

**Methods:**

Microarray datasets were obtained from Gene Expression Omnibus (GEO) database. Principal component analysis (PCA) and immune infiltration analysis were performed to select suitable samples for further analysis. Differential analysis and functional enrichment analysis were conducted on DC samples, comparing live MTB-infected and non-infected (NI) groups. The CytoHubba plugin in Cytoscape was used to identify hub genes from the differentially expressed genes (DEGs). The expression of the hub genes was validated using two datasets and reverse transcription-quantitative polymerase chain reaction (RT-qPCR) in human monocyte-derived DCs. Enzyme-linked immunosorbent assay (ELISA) was used to validate interferon (IFN) secretion. Transcription factors (TFs) and microRNAs (miRNAs) that interact with the hub genes were predicted using prediction databases. The diagnostic value of the hub genes was evaluated using receiver operating characteristic (ROC) curves and area under the curve (AUC) values.

**Results:**

A total of 1835 common DEGs among three comparison groups (18 h, 48 h, 72 h after MTB infection) were identified. Six DEGs (*IFIT1*, *IFIT2*, *IFIT3*, *ISG15*, *MX1*, and *RSAD2*) were determined as hub genes. Functions enrichment analysis revealed that all hub genes all related to IFN response. RT-qPCR showed that the expression levels of six hub genes were significantly increased after DC stimulated by live MTB. According to the results of ELISA, the secretion of IFN-γ, but not IFN-α/β, was upregulated in MTB-stimulated DCs. AUC values of six hub genes ranged from 84 to 94% and AUC values of 5 joint indicators of two hub genes were higher than the two hub genes alone.

**Conclusion:**

The study identified 6 hub genes associated with IFN response pathway. These genes may serve as potential diagnostic biomarkers in tuberculosis (TB). The findings provide insights into the molecular mechanisms involved in the host immune response to MTB infection and highlight the diagnostic potential of these hub genes in TB.

**Supplementary Information:**

The online version contains supplementary material available at 10.1186/s12920-023-01646-0.

## Introduction

Tuberculosis (TB) is a global communicable disease caused by *Mycobacterium tuberculosis* (MTB) and remains a major cause of illness and death worldwide before the coronavirus disease 2019 (COVID-19) pandemic [1]. The mammalian host defense against MTB relies on both innate and adaptive immunity [2, 3]. Dendritic cells (DCs) serve as a crucial link between innate and adaptive immune responses [4]. In peripheral tissues, immature DCs act as sentinels, sensing the presence of MTB and initiating an inflammatory response. They can sense the presence of MTB in the airway and engulf bacteria, which assist in the induction of a rapid inflammatory response and accumulation of immune cells [5]. Furthermore, DCs are the most powerful antigen-presenting cells (APCs) and are essential for the priming of adaptive immune responses in *vivo* [6]. After being stimulated by MTB, immature DCs begin to turn into a mature state, including expression of C-C chemokine receptor 7 (CCR7), up-regulation of MHC class II molecules, and cluster of differentiation (CD) 80, CD86, and CD40 costimulatory molecules [7]. Simultaneously, DCs migrate to the draining lymph nodes and then secrete inflammatory mediators that drive the activation of the T lymphocytes differentiation which are necessary to clear MTB infection [4]. DCs play an important role in the host defense against MTB, but the interactions between MTB and DCs are not fully understood. By unraveling the intricate interactions between DCs and MTB, researchers can enhance our understanding of the immune response to TB and potentially identify novel targets for therapeutic interventions, vaccine development, and diagnostic strategies.

Early and accurate diagnosis is essential for controlling the spread of TB. The GeneXpert MTB/RIF assay is a rapid diagnostic test for detection of TB and rifampicin resistance recommended by World Health Organization (WHO) [8]. However, the high price of GeneXpert MTB/RIF assay limits the application. there is a need for affordable and suitable tools for TB screening and diagnosis. Biomarkers are emerging as potential alternative tools for TB diagnosis, prediction of disease progression, and evaluation of treatment efficacy [9], such as interferon-γ (IFN-γ) release assay (IGRA) for identification of latent tuberculosis infection (LTBI). Recent transcriptomic studies have identified biomarkers expressed by monocytes, including monocyte-derived DCs, in the context of MTB infection [10]. These biomarkers provide insights into the immune response and can potentially be utilized for TB diagnosis and monitoring.

By exploring and validating these biomarkers, researchers aim to develop affordable and accessible diagnostic tools for TB. These biomarkers have the potential to enhance early detection, improve patient management, and contribute to TB control efforts in resource-limited settings. Efforts are underway to identify additional biomarkers and develop cost-effective diagnostic approaches that can be easily implemented in low-income areas, thereby improving TB diagnosis and reducing the burden of the disease. In this study, we identified six hub genes in DCs during MTB infection using integrated bioinformatics analysis and the results were further confirmed in human monocyte-derived DCs, which provide an insight into the process of activated DCs against infection with MTB and explore potential biomarkers for early diagnosis of TB.

## Materials and methods

### Data preparation

We obtained two gene expression microarray datasets from Gene Expression Omnibus (GEO) database (http://www.ncbi.nlm.nih.gov/geo). GSE116405 [11] dataset was obtained from GPL16791 platform. Blood mononuclear cells were isolated by Ficoll-Paque centrifugation and blood monocytes were purified from peripheral blood mononuclear cells (PBMCs) by positive selection with magnetic CD14 MicroBeads (Miltenyi Biotec). Monocytes were then derived into DCs. The number of samples in non-infected (NI), live MTB-infected, and heat-inactivated (HI) MTB-infected group were 17, respectively, at multiple time points in hours (h), 2 h, 18 h, 48 h, and 72 h. DCs were infected with MTB for time series at a multiplicity of infection (MOI) of 1 or with HI MTB at MOI of 5. GSE163531 [12] dataset was obtained from GPL20265 platform and 8 DC samples from blood were treated with or without virulent MTB H37Rv (ATCC 27,294) at MOI of 1 bacterium/cell for 16 h. DCs were generated by culturing CD14 + monocytes with 50 ng/ml granulocyte-macrophage colony-stimulating factor (GM-CSF) and 1000 U/ml IL-4. Detailed information about these datasets were listed in Table 1.

**Table 1:** Details of GEO Datasets

Accession	Platform	Experiment Type	Treatment	Time(h)	number of replicates	Cell Type
GSE116405	GPL16791	Expression profiling by high throughput sequencing	HI MTB (5)	2	4	Monocyte-derived dendritic cells
			HI MTB (5)	18	5	
			HI MTB (5)	48	5	
			HI MTB (5)	72	3	
			live MTB (1)	2	4	
			live MTB (1)	18	5	
			live MTB (1)	48	5	
			live MTB (1)	72	3	
			NI	2	4	
			NI	18	5	
			NI	48	5	
			NI	72	3	
GSE163531	GPL20265	Expression profiling by array	live MTB (1)	16	4	Primary dendritic cells
			NI	16	4	

Abbreviations: GEO, Gene Expression Omnibus; HI, heat inactivation; MTB, Mycobacterium tuberculosis, NI, non-infected; MOI, multiplicity of infection. Note: Numbers in brackets represent the MOI in that group

### Principal component analysis (PCA) and Immune infiltration analysis

PCA was calculated using FactoMineR (https://cran.r-project.org/web/packages/FactoMineR/index.html) and factoextra (https://cloud.r-project.org/package= factoextra/). R package was used to generate PCA plot that can distinguish the control and experimental samples. PCA allows for the reduction of high-dimensional data and provides an overview of the variability in the dataset. The generated PCA plot helps distinguish between control and experimental samples based on their gene expression profiles. Clustering of samples together indicates that low variability among those samples, while greater separation suggests higher variability. CIBERSORT algorithm [13] was utilized for estimation of the proportion of immune cells. The algorithm uses gene expression data to infer the composition of immune cell types within a sample. Standard RNA-Seq expression quantification metrics of the GSE116045 dataset, transcripts per kilobase million (TPM)-normalized expression levels, were evaluated with immune infiltration analysis using the Cibersort R script (https://rdrr.io/github/singha53/amritr/src/R/supportFunc_cibersort.R) and the LM22 signature gene file of *22* immune cell types [13]. By utilizing these methods, you aimed to gain insights into the immune cell composition and understand the variability in gene expression profiles between control and experimental samples. The PCA plot and CIBERSORT analysis provide valuable information for further exploration and interpretation of the dataset.

### Differentially expressed genes (DEGs) analysis

DEGs analysis was performed using the DEseq2 package [14] in R on count data. The parameters, |log_2_FC| > 1.5 (absolute log2 fold change greater than 1.5) and adj.P.Value < 0.05 (adjusted p-value less than 0.05), were used as the screening criteria for DEGs based on the results of immune infiltration analysis on the GSE116405 dataset. After comparisons of live MTB-infected samples vs. NI samples at three time points (18 h, 48 h, and 72 h), DEGs among three respective groups were identified by the parameters that determine their common DEGs for further analysis. Additionally, the ComplexHeatmap [15] package in R was used to generate the expression heatmap. Heatmaps provided a visual representation of gene expression patterns across samples, allowing for the identification of clusters and patterns of upregulation or downregulation. The volcano plots of three groups were drawn with R package ggplot2. By following these steps, you conducted DEG analysis, identified common DEGs among the three groups, and visualized the gene expression patterns using a heatmap and volcano plots. These analyses provided insights into the genes that were differentially expressed in response to MTB infection at different time points, facilitating further investigation of their roles and functions in the immune response to TB.

### PPI (protein–protein interaction) network construction and hub genes identification

STRING (https://string-db.org/), a prediction database dedicated to protein-protein interactions, was used to search for interactions between DEGs and known proteins. The identified common DEGs were uploaded to STRING’s official website for evaluating the PPI networks interactive relationships among DEGs which were determined based on a high confidence score threshold set to 0.90. Subsequently, PPI networks including nodes and edges with protein-protein interactions were visualized using Cytoscape [16] (version 3.7.0). CytoHubba [17], a Cytoscape plugin, was used to explore PPI networks hub genes with top ten ranking scores based on a maximal clique centrality (MCC) algorithm [17].

### Functional enrichment analysis of DEGs

Clusterprofiler [18] R package was used here to perform functional enrichment analysis of the common DEGs including gene ontology (GO, http://geneontology.org) function analysis to obtain function-related terms and Kyoto Encyclopedia of Genes and Genomes (KEGG) [19] pathway analysis to obtain functional pathway-related information. The GO terms were grouped into three categories of biological process (BP), cellular component (CC), or molecular function (MF). Adj.P.Value < 0.05 was considered statistically significant in GO terms and KEGG pathways.

### Validation of hub genes

To validate the accuracy of the obtained key genes, the expressions of hub genes were identified using GSE16351 datasets and RT-qPCR assays. PBMCs were isolated from the EDTA-treated blood of 4 healthy and 8 TB individuals by Ficoll-Hypaque density gradient centrifugation. Isolated PBMCs were cultured in RPMI 1640 containing 10% fetal bovine serum (FBS), 4 mM L-glutamine, and 1% penicillin-streptomycin (Invitrogen), 10ng/ml recombinant human IL4 (rhIL-4, Cat Z02925, GenScript) were added into the culture, and 50ng/ml of recombinant human granulocyte-macrophage colony-stimulating factor (rhGM-CSF, Cat Z02695; GenScript) for 5 days at 37℃with 5% CO2 to allow the differentiation of monocytes into DCs. Monocyte-derived dendritic cells (Mo-DCs) were seeded in 6- well plates at a density of 1 × 10^6^ cells per well. MTB H37Rv strains were cultured on Löwenstein-Jensen (L-J) slants for 3weeks at 37℃ to mid-logarithmic phase and scraped. MTB was collected adequately ground and resuspended using cell culture medium with 0.05% Tween-80. After resting for 15 min, the upper layer of the bacterial suspension was taken and adjusted to OD600 = 1, which value corresponds to 1 × 10^8^ colony-forming units (CFUs)/ ml of MTB. Mo-DCs were then infected with MTB at MOI of 1 or with heat-killed MTB at MOI of 5:1 for 24 h at 37℃. For detection of *IFIT1*, *IFIT2*, *IFIT3*, *ISG15*, *MX1*, and *RSAD2* mRNAs, total RNA extraction of Mo-DCs was carried out using a General RNA Extraction Kit (Vazyme Biotech). The reverse-transcription of RNA was accomplished by using a 1st Strand cDNA Synthesis SuperMix (TransGen Biotech) and performed quantitative RT-PCR (qPCR) analysis with qPCR SYBR Green Master Mix (Foreverstar Biotech) on Applied Biosystems 7500 Real-Time PCR system. Data were analyzed using the 2−^Δ^^∆CT^ method with normalization to the expression of the control gene GAPDH. All qPCR primers were listed in Table S1. The paired t-test was applied for statistical verification of two online expression data and experimental data. *P*-value < 0.05 was defined as statistically significant. The protocol was approved by the Ethics Committee of Beijing Chest Hospital, Capital Medical University.

For enzyme-linked immunosorbent assay (ELISA), the culture media were collected and sterile-filtered through a 0.22-μm filter (Millipore) after 24 h post-infection. Human IFN-α ELISA kit (cat#EHC144a, Xinbosheng Biotech), Human IFN-β ELISA kit (cat#EHC026B, Xinbosheng Biotech), Human IFN-γ ELISA kit (cat#EHC102g, Xinbosheng Biotech) was used for ELISA according to the manufacturer’s instructions.

### The operating characteristic (ROC) curves of hub genes

ROC curves and area under the curve (AUC) values were calculated to evaluate the diagnostic value of the six hub genes using the pROC [20] R language package. To analyze the combined index of two hub genes randomly in six, we constructed a logistic regression and predict variables named “Probe” using the glmnet [21] R package. Three ROC curves and AUC values were plotted separately to compare the predictive power of the two hub genes and their combined indexes.

### Analysis of microRNAs (miRNAs) and transcription factors (TFs) Interaction Network of the six hub genes

To further explore the relationship between hub genes and miRNA, the miRWalk database (http://mirwalk.umm.uni-heidelberg.de/) [22] was used to predict the target genes of miRNA. For key genes interaction with transcription factor analysis, we correspondingly explored transcription factor based on ChEA3 (https://maayanlab.cloud/chea3/) [23] prediction database. Additionally, the established mutual relationship data were imported into Cytoscape software for network visualization.

## Results

### Screening samples based on PCA and immune infiltration analysis

A total of 51 samples were divided into four groups by four time points and then into three groups, NI group, live MTB-infected group, and HI MTB-infected group, based on different treatments. The results of the PCA were shown in Figure S1. In the GSE116405 dataset, the PCA plot (Figure S1A) showed that both the live MTB-infected group and HI MTB-infected group clustered together, indicating no significant difference between them. As shown in Figure S1B, three time point groups (18 h, 48 h, and 72 h) clustered together, suggesting that there was no significant difference among these groups. Neither the NI group nor the 2 h group clustered with the other groups, indicating that they had distinct expression patterns compared to the other groups.

**Fig. 1:**
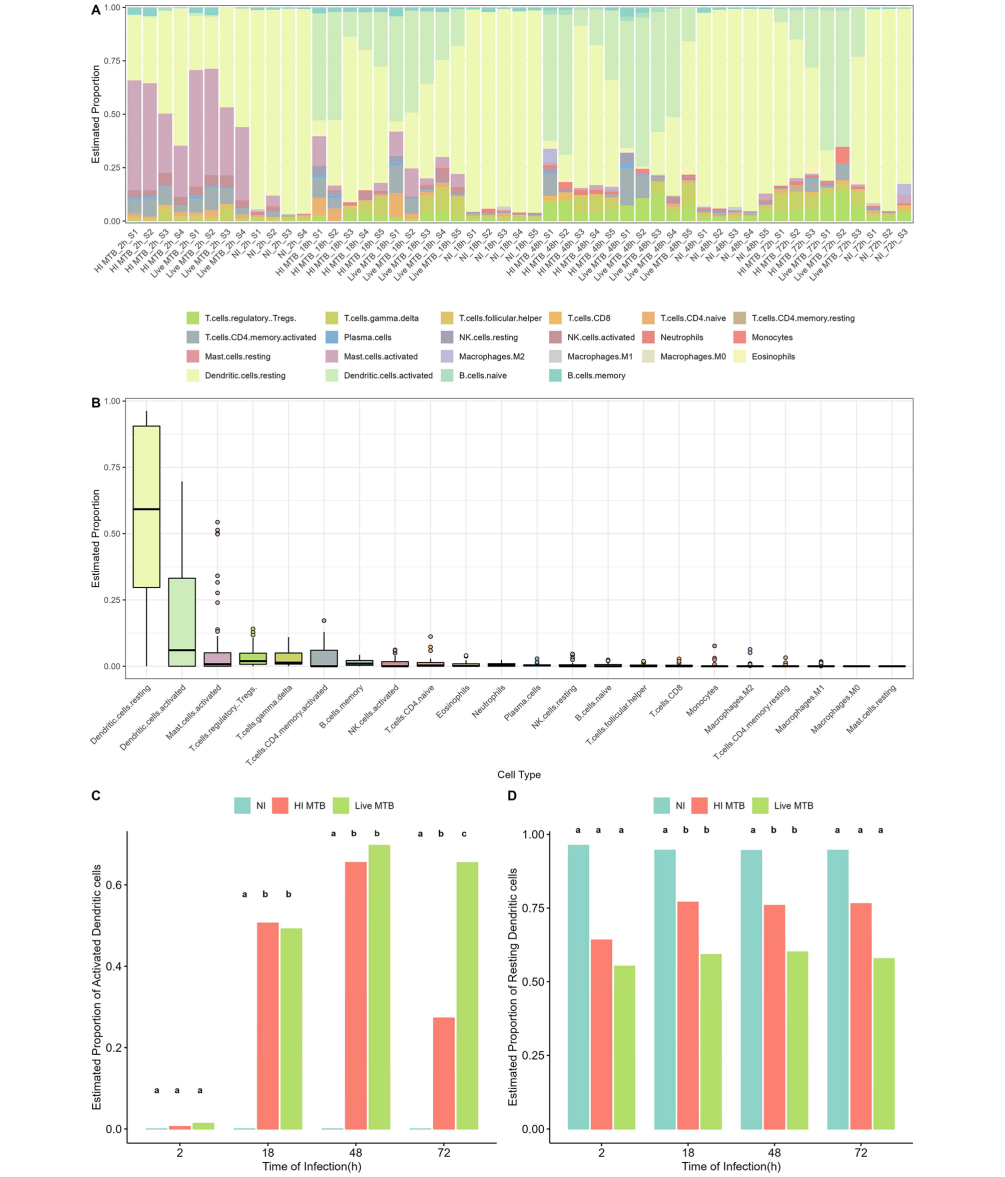
Screening samples using immune infiltration analysis. **(A)** Relative proportions of the 22 immune cell types of 52 samples in GSE116405 dataset. **(B)** Boxplots displaying all estimated proportion values of each immune cell type in 52 samples were plotted and sorted in descending order by the means. **(C)** The bar graphs represent summarized all estimated proportion values of activated dendritic cells in samples for four time points in each group. **(D)** The bar diagrams about resting dendritic cells were summarized as in **(C)**. (Significant differences between the groups are indicated by different superscript letters (a, b, and c) (p < 0.05); no significant differences between the groups are indicated by same superscript letters (p > 0.05).)

The estimated proportion of immune cells using the CIBERSORT algorithm showed that resting and activated DCs had the highest estimated proportion among the 22 types of immune cells in most of the samples (Fig. 1A). The estimated proportion values of resting or activated DCs were generally higher than other immune cells (Fig. 1B), indicating the importance of DCs in the immune response. The estimated proportion values of activated DCs in the live MTB-infected group were similar to those in the HI MTB-infected group from 2 to 48 h except 72 h (Fig. 1C). Both of live or HI MTB-infected group had statistically significant differences compared with the NI group from 2 to 72 h (Fig. 1C). NI group had higher estimated proportion values of resting DCs compared to live MTB-infected group and HI MTB-infected group (Fig. 1D). HI MTB-infected group had higher estimated proportion values of resting DCs compared to live MTB-infected group, especially at 18 and 48 h. The resting DCs was the majority of immune cells in the NI group.

Therefore, DCs infected with MTB for 18 h, 48 h, and 72 h had a better performance on DC activation levels than 2 h and it was observed with a significant difference between the NI group and MTB-infected group using the PCA. These findings suggest that the activation levels of DCs were influenced by MTB infection and vary across different time points. The PCA analysis and immune infiltration analysis provided valuable insights into the dynamics of DC activation in response to MTB infection.

### Identification of DEGs

**Fig. 2:**
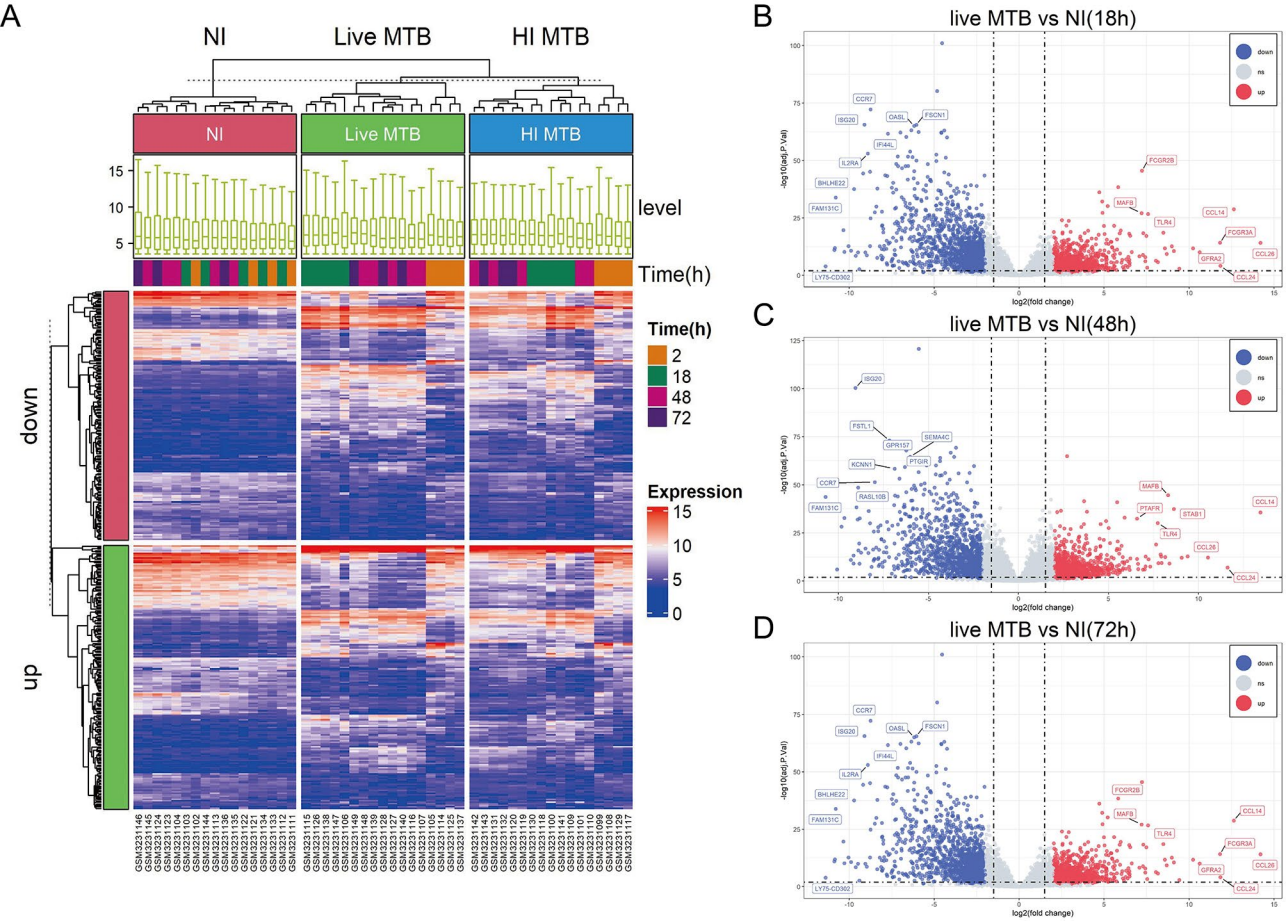
Heatmap and volcano analysis of differentially expressed genes for the screened GSE116405 dataset. **(A)** A total of 325 DEGs divided into up- and down-regulated genes represent their expression level blue indicates a low level and red is the opposite. Volcano plot shows standard-compliant (adj.P.Value < 0.05 and |log2 (FC)|≥1.5) DEGs from live MTB vs. NI group for 18 h **(B)**, 48 h **(C)** and 72 h **(D)**. (DEGs, differentially expressed genes; FC, fold change; MTB, *Mycobacterium tuberculosis*; NI, non-infected)

Differential analysis was performed using above screened samples of the GSE116405 dataset and its gene expression profiles. In live MTB vs. NI group, 708 up- and 1145 down-regulated, 938 up- and 1170 down-regulated, and 1014 up- and 1327 down-regulated DEGs were identified by samples from 18, 48, and 72 h, respectively. The heatmap of top significantly 325 DEGs in total including top 100 up- and top 100 down-regulated DEGs in each group were visualized in Fig. 2A. Volcano plots show DEGs between the live MTB-infected and NI groups for 18 h, 48 h, and 72 h (Fig. 2B-D).

### PPI Network Construction and Selection of Hub Gene

**Fig. 3:**
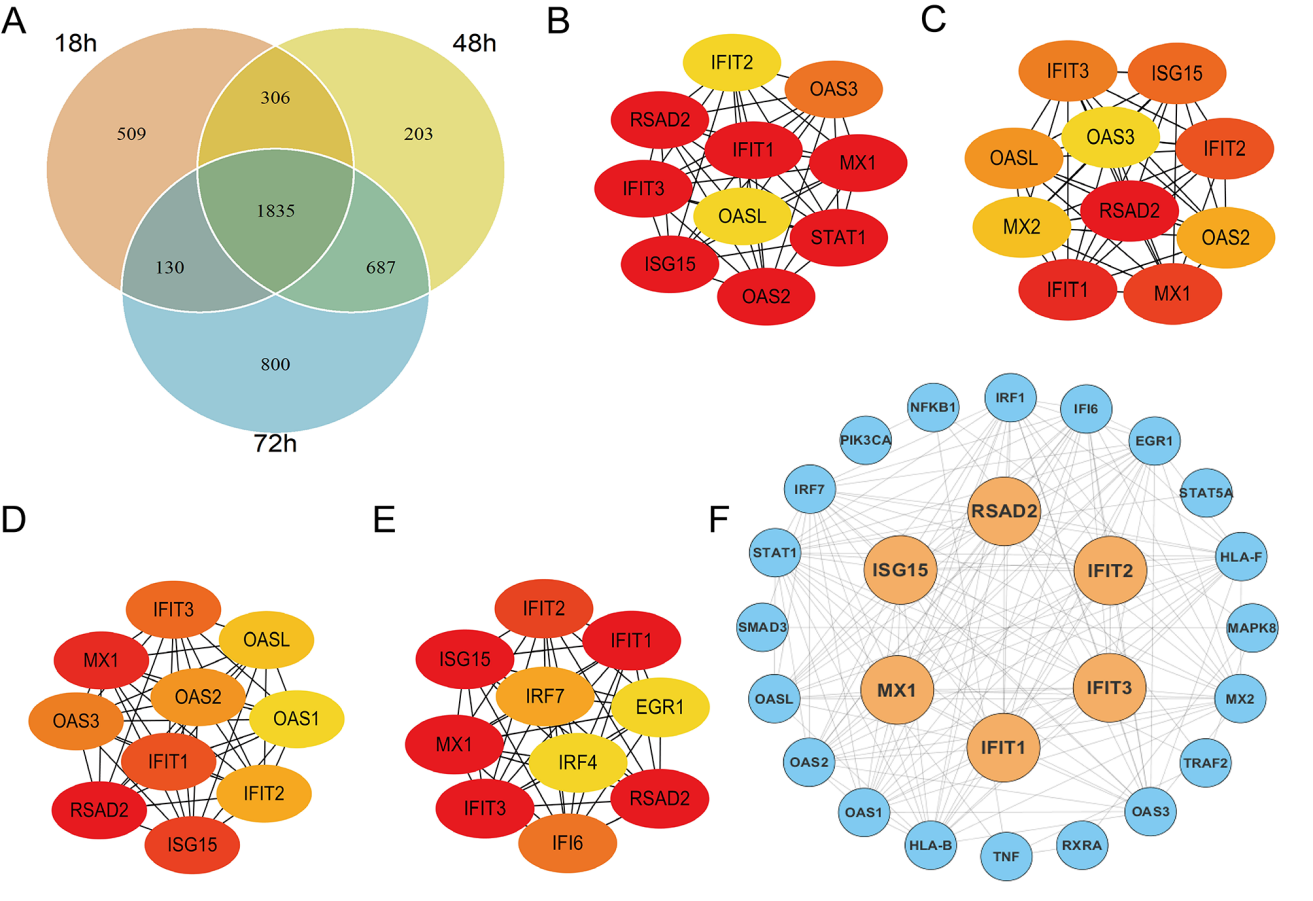
PPI Network Construction and Selection of Hub Gene. **(A)** Venn diagram comparison analysis for DEGs of 18 h, 48 h, and 72 h on live MTB-infected vs. NI group. **(B)** Identification of ten hub genes in the 18 h live MTB-infected group compared with 18 h NI group using the plugin cytoHubba of Cytoscape evaluated with MCC scores. **(C)** Identification of ten hub genes in the 48 h live MTB-infected group compared with 48 h NI group using the plugin cytoHubba of Cytoscape evaluated with MCC scores. **(D)** Identification of ten hub genes in the 72 h live MTB-infected group compared with 72 h NI group using the plugin cytoHubba of Cytoscape evaluated with MCC scores. **(E)** Identification of ten hub genes in 1835 common DEGs using the plugin cytoHubba of Cytoscape evaluated with MCC scores. **(F)** PPI network construction on 1835 common DEGs was visualized by Cytoscape software. (PPI, protein-protein interactions; MTB, *Mycobacterium tuberculosis*; NI, non-infected; DEGs, differentially expressed genes; MCC, maximal cinque centrality)

Venn diagram showed the intersection results of DEGs among three groups above, in which there are 1835 common DEGs (Fig. 3A). DEGs for three time points and their intersection were further analyzed on STRING database and the top 10 key genes of DEGs from three time points and their intersection were identified using the plug-in cytoHubba of Cytoscape and ranking by MCC scores (Table S1 and Fig. 3B-E). 9 common key genes occured in all top 10 key genes of three time points which were depicted in Table S1. Then, six key genes (*IFIT1*, *IFIT2*, *IFIT3*, *ISG15*, *MX1*, and *RSAD2*), all appearing in the four groups of Table S1, were selected as hub genes (Table 2). Finally, six hub genes were visualized in the PPI network of 1835 common DEGs that was composed of 553 nodes and 1989 edges and its nodes with MCC high scores are presented in Fig. 3F.

**Table 2:** Six Hub Genes

Gene symbol	Type	Gene Title	Pathway Involved	GO Term Involved
IFIT1	Upregulation	Interferon induced protein with tetratricopeptide repeats 1	Hepatitis C	response to virus, defense response to virus, defense response to symbiont, regulation of response to biotic stimulus, response to type I interferon, cellular response to type I interferon, negative regulation of protein binding, etc.
IFIT2	Upregulation	Interferon induced protein with tetratricopeptide repeats 2	/	response to virus, defense response to virus, negative regulation of protein binding, regulation of binding
IFIT3	Upregulation	Interferon induced protein with tetratricopeptide repeats 3	/	response to virus, response to interferon-alpha, defense response to virus, response to interferon-alpha, defense response to symbiont
ISG15	Upregulation	ISG15 ubiquitin like modifier	Epstein-Barr virus infection, RIG-I-like receptor signaling pathway, Human papillomavirus infection	regulation of interferon-gamma production, interferon-gamma production, response to virus, negative regulation of immune system process, regulation of hemopoiesis, defense response to symbiont, regulation of response to biotic stimulus, regulation of innate immune response, etc.
MX1	Upregulation	MX dynamin like GTPase 1	Measles, Influenza A, Human papillomavirus infection, Hepatitis C	response to virus, defense response to virus, defense response to symbiont, negative regulation of viral process, viral process, regulation of viral process, regulation of viral life cycle, viral genome replication, etc.
RSAD2	Upregulation	Radical S-adenosyl methionine domain containing 2	Influenza A, Hepatitis C	type 2 immune response, response to virus, positive regulation of cytokine production, mononuclear cell differentiation, defense response to symbiont, T cell differentiation, negative regulation of viral genome replication, regulation of immune effector process, CD4-positive alpha-beta T cell differentiation, regulation of adaptive immune response, etc.

Abbreviation: GO, Gene Ontology

### GO functional and KEGG enrichment analysis of common DEGs

**Fig. 4:**
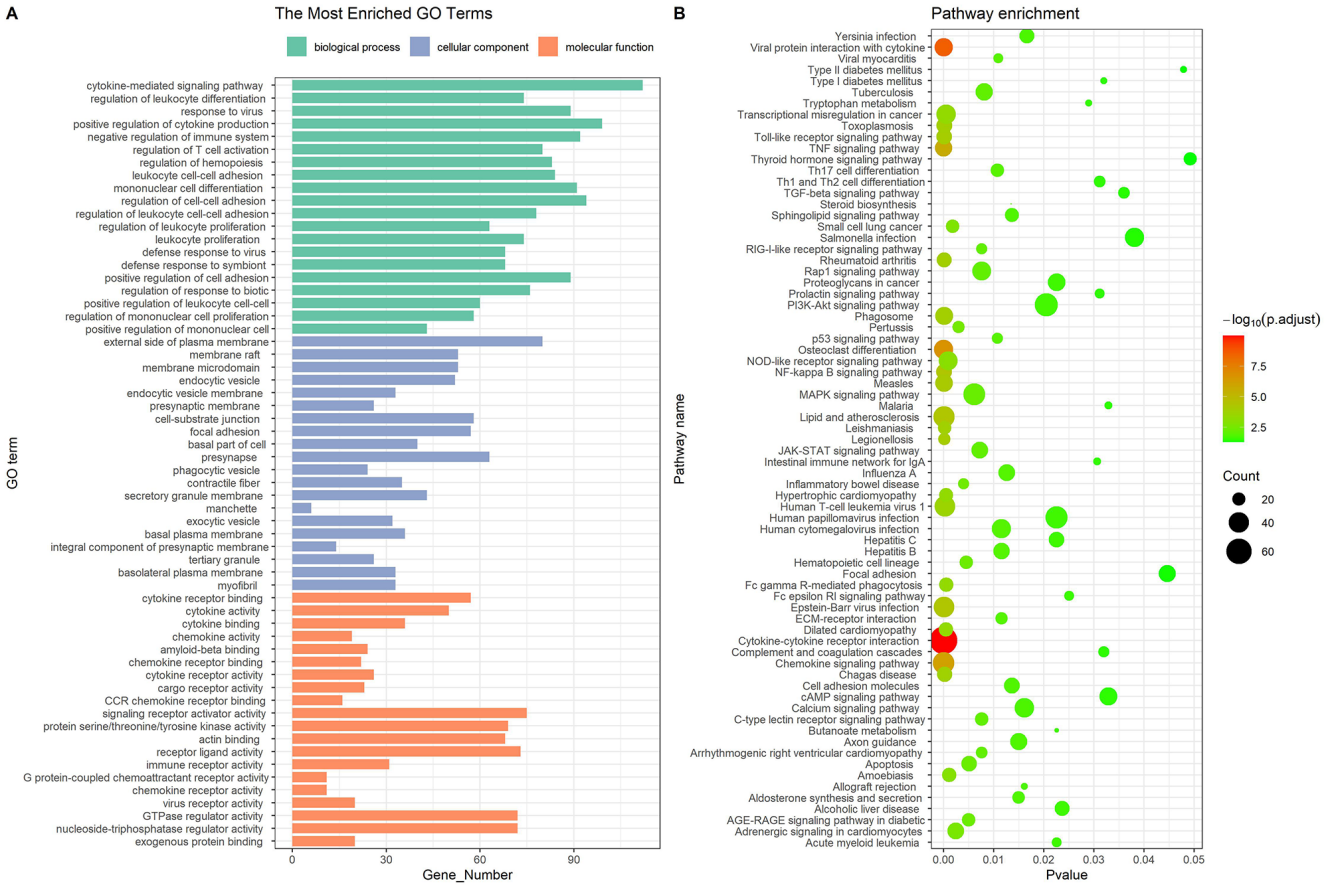
Functional enrichment analysis of common genes shared in the three sets of DEGs. **(A)** Common DEGs were enriched in three categories of GO function including BP, CC, and MF. **(B)** The results of KEGG pathway enrichment were shown in bubble diagram. (DEGs, differentially expressed genes; GO, gene ontology; CC, cellular component; MF, molecular function; BP, biological process; KEGG, Kyoto Encyclopedia of Genes and Genomes)

To further explore the molecular mechanism during MTB infection of DCs, GO enrichment analysis and KEGG pathway analysis based on common DEGs were performed using clusterProfiler method (Fig. 4). The top 20 enriched BP terms included cytokine-mediated signaling pathway, regulation of leukocyte differentiation and response to virus, among others. These findings suggest that MTB infection in DCs may lead to the activation of cytokine signaling pathways, modulation of leukocyte differentiation, and response to infection. The top 20 enriched MF terms included cytokine receptor binding, cytokine activity, and cytokine binding, etc. This indicates that the DEGs may be involved in interactions with cytokine receptors and exhibit cytokine-related activities. The top 20 enriched CC terms included external side of plasma membrane, membrane raft, and membrane microdomain, etc. These results suggest that the DEGs may be associated with specific membrane locations involved in immune responses. KEGG pathway enrichment results were significantly enriched for cytokine-cytokine receptor interaction, viral protein interaction with cytokine and cytokine receptor, osteoclast differentiation, chemokine signaling pathway, and TNF signaling pathway, among others. These findings indicate that the DEGs are involved in various immune-related pathways. More details of the enrichment analysis containing GO and KEGG are shown in Table S2&S3. All hub genes identified in the study were found to be enriched in pathways or GO terms related to immune responses, particularly those associated with IFN (Table 2). This suggests that these hub genes are involved in IFN-mediated immune responses during MTB infection in DCs. These results indicate that MTB infection in DCs triggers a wide range of immune responses and signaling pathways. The enrichment analysis provides valuable insights into the molecular mechanisms underlying the immune response to MTB infection in DCs, with a particular emphasis on the role of IFN and its associated pathways.

### Validation of hub gene expression and IFN Release

**Fig. 5:**
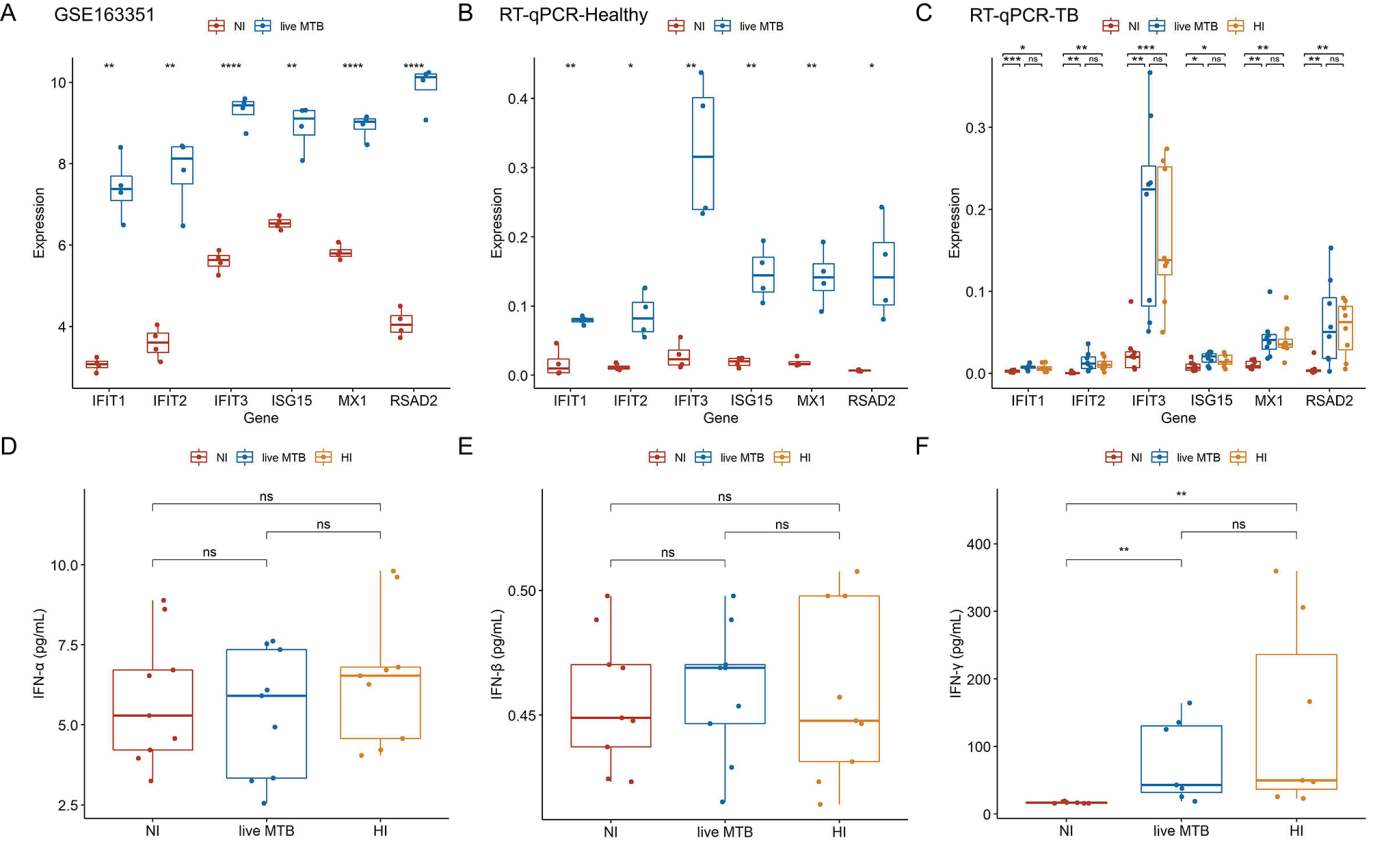
Validation of expression levels of 6 hub genes in MTB-infected DCs. Based on **(A)** GSE163351, there is a statistically significant difference between MTB-infected and NI DCs in six hub gene expression levels. The statistically significant results of expression levels from healthy populations **(B)** and TB populations **(C)** validated by RT-qPCR were displayed in a boxplot. IFN-α/β **(D-E)** and IFN-γ **(F)** secretion quantified by ELISA were presented in boxplots. Paired t-test was used to compare the means of the control and the treated groups (*: *p* < 0.05, **: *p* < 0.01, ***: *p* < 0.001, ****: *p* < 0.0001, *ns*.: no significance). (MTB, *Mycobacterium tuberculosis*; NI, non-infected; DCs, dendritic cells; TB, tuberculosis; RT-qPCR, reverse transcription-quantitative polymerase chain reaction; Mo-DCs, Monocyte-derived dendritic cells; IFN, interferon; ELISA, enzyme-linked immunosorbent assay)

To further confirm the selected six hub genes, we compared the expression levels of six selected hub genes using transcriptomic data from GSE163531 (Fig. 5A) dataset. Subsequently, 4 healthy volunteers were enrolled to isolate human PBMCs (Table S5) and Mo-DCs were infected by MTB H37Rv. RT-qPCR was used to determine the gene expression levels, in which all six hub genes had statistically significant differences between NI and MTB infection groups (Fig. 5B). Samples of 8 TB patients used for RT-qPCR also indicated results of significant differences between NI, live and HI MTB infection groups (Fig. 5C). These results confirmed that the mRNA expression level of six hub genes in DCs originating from healthy and TB populations will be significantly increased after stimulation by MTB. The enrichment analysis results and these hub genes all were strongly related to type I IFN (IFN-α/β) and type II IFN (IFN-γ). To validate the release of these IFNs, ELISA experiments were performed. The results showed that the secretion of IFN-γ, but not IFN-α/β, was upregulated in MTB-stimulated DCs (Fig. 5D-F). This observation further supports the involvement of IFN in the immune response to MTB infection. Overall, the expression analysis, including both transcriptomic data and RT-qPCR validation, confirms the significant upregulation of the six hub genes in DCs upon MTB stimulation. Additionally, the ELISA experiments validated the release of IFN-γ in response to MTB infection, supporting the association between the hub genes and IFN-γ signaling. These findings provide further evidence of the involvement of these hub genes and IFN-γ in the immune response to MTB infection in DCs.

### ROC curves analysis of the six hub genes

**Fig. 6:**
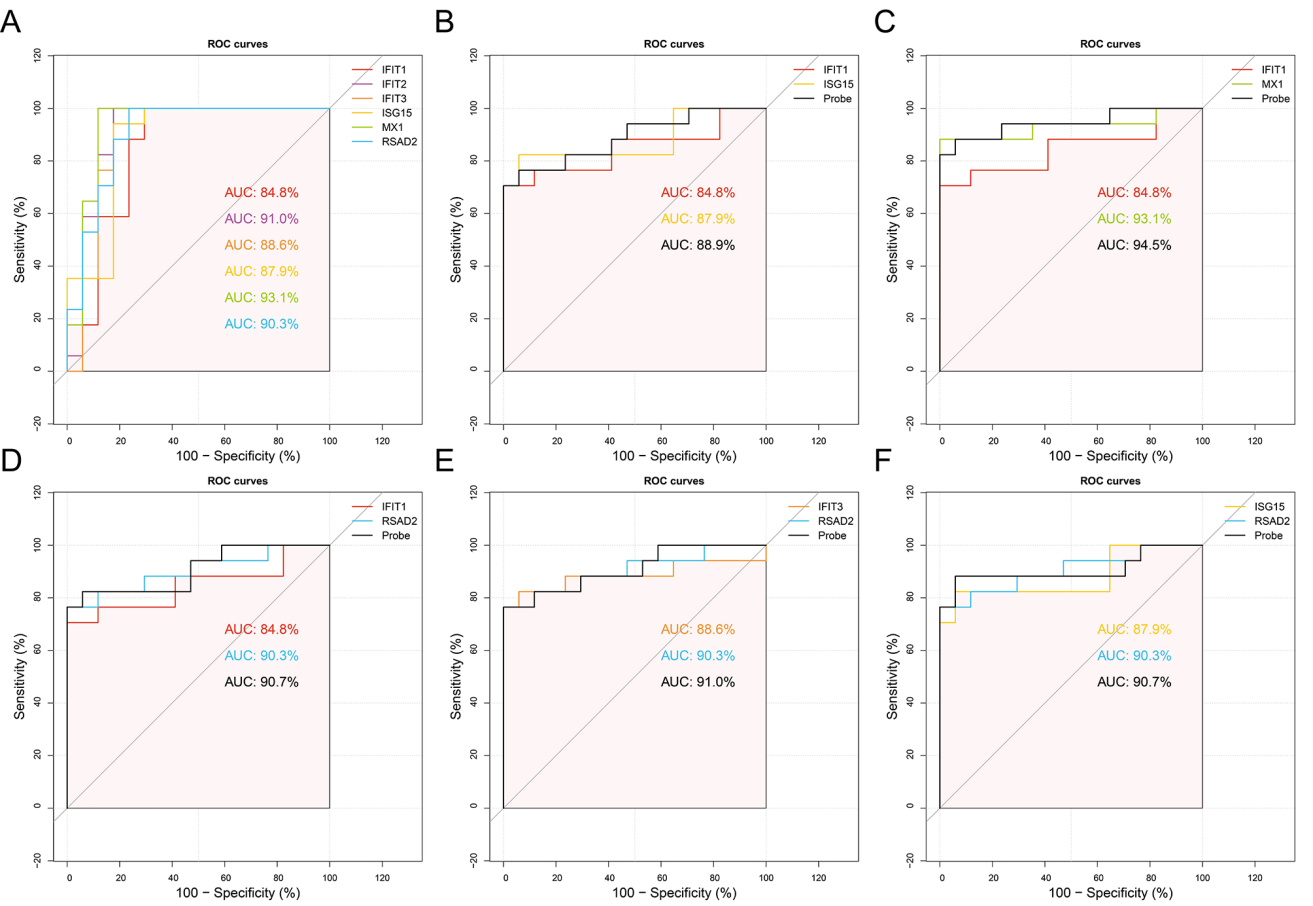
ROC curves for diagnostic evaluation of the six hub genes. **(A)** ROC curves and AUC of six hub genes (*IFIT1*, *IFIT2*, *IFIT3*, *ISG15*, *MX1*, and *RSAD2*). Five improved AUC values of combined diagnostic “Probe” were generated from two of the six hub genes including **(B)***IFIT1* and *ISG15*, **(C)***IFIT1* and *MX1*, **(D)***IFIT1* and *RSAD2*, **(E)***IFIT3* and *RSAD2*, and **(F)***ISG15* and *RSAD2*. (ROC, receiver operating characteristic; AUC, area under the ROC curve)

The ROC curves and AUC analysis were performed to further investigate the diagnostic value of the six MTB infection-related hub genes in DCs, (Fig. 6). Based on GSE116405 datasets, the AUC values of six hub genes *IFIT1* (AUC = 84.4%), *IFIT2* (AUC = 91.0%), *IFIT3* (AUC = 88.6%), *ISG15* (AUC = 87.9%), *MX1* (AUC = 93.1%), and *RSAD2* (AUC = 90.3%) (Fig. 6A). Next, we randomly selected two of the six hub gene expression levels in above dataset to construct a logistic regression model which generated a variable named “Probe” and included the predicted probability value of the joint indicator. Three ROC curves were plotted to compare the diagnostic value of the two hub genes separately and their combined indicator “Probe” with each other. Figure 6B-F, there are five “Probe” AUC values in our analysis that are higher than the two hub genes that displayed the results of the combined diagnostic value of these two indicators containing *IFIT1* and *ISG15* (AUC = 88.9%), *IFIT1* and *MX1* (AUC = 94.5%), *IFIT1* and *RSAD2* (AUC = 90.7%), *IFIT3* and *RSAD2* (AUC = 91.0%) and *ISG15* and *RSAD2* (AUC = 90.7%), of which the “Probe” AUC values were higher than the two hub genes alone. In summary, all AUC values generated from our ROC curves analysis ranged from 84 to 95%, which indicates that these six hub genes may be potential diagnostic biomarkers in TB.

### Construction of miRNA and TFs Interaction Network of the six hub genes

**Fig. 7:**
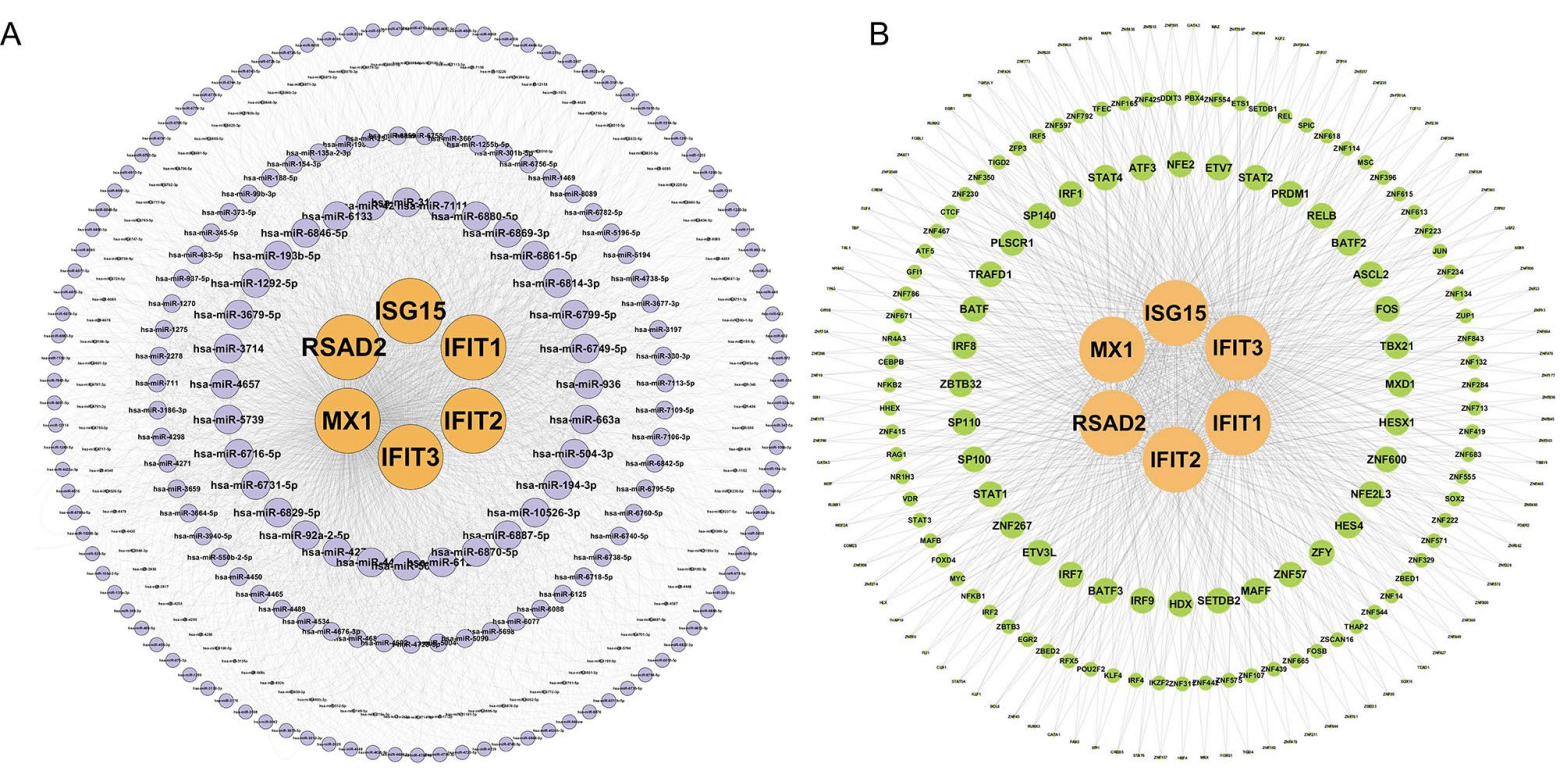
Prediction and construction of miRNA-hub gene **(A)** and TF-hub gene **(B)** interaction networks. (miRNA, microRNA; TF, transcription factor)

To evaluate the related miRNA and TFs which may regulate the six hub genes expression, miRWalk prediction database and ChEA3 database were used to construct interaction networks between miRNA or TFs with the six hub genes, respectively (Fig. 7A and B). The predicted miRNA with a high number of gene cross-links (≥ 4) are listed in Table 3. We also conducted a comparison analysis between the up-regulated DEGs from three time points and predicted TFs. Several related TFs were found significantly changed after MTB infection (Table 4), which indicated that these TFs may participate in the regulation of expression levels of these six hub genes.

**Table 3:** Predicted miRNAs and at least 4 hub genes targeted by the same miRNA

miRNA		Genes targeted by miRNA		Gene count
hsa-miR-5196-5p		IFIT3, MX1, IFIT1, IFIT2, RSAD2		5
hsa-miR-6749-5p		IFIT3, MX1, IFIT1, IFIT2, RSAD2		5
hsa-miR-6799-5p		IFIT3, MX1, IFIT1, IFIT2, RSAD2		5
hsa-miR-885-3p		IFIT3, IFIT1, IFIT2, RSAD2		4
hsa-miR-936		IFIT3, MX1, IFIT1, IFIT2		4
hsa-miR-3137		IFIT3, MX1, IFIT1, RSAD2		4
hsa-miR-3677-3p		IFIT3, MX1, IFIT1, IFIT2		4
hsa-miR-4685-3p		IFIT3, IFIT1, IFIT2, RSAD2		4
hsa-miR-6086		IFIT3, MX1, IFIT1, IFIT2		4
hsa-miR-6813-5p		IFIT3, IFIT1, IFIT2, RSAD2		4
hsa-miR-6848-5p		IFIT3, IFIT1, IFIT2, RSAD2		4
hsa-miR-6860		IFIT3, IFIT1, IFIT2, RSAD2		4
hsa-miR-6869-3p		IFIT3, MX1, IFIT1, RSAD2		4
hsa-miR-6872-3p		IFIT3, IFIT1, IFIT2, RSAD2		4
hsa-miR-7106-5p		IFIT3, IFIT1, IFIT2, RSAD2		4
hsa-miR-7111-5p		IFIT3, MX1, IFIT2, RSAD2		4
hsa-miR-9851-5p		IFIT3, MX1, IFIT1, RSAD2		4
hsa-miR-1976		IFIT3, IFIT1, IFIT2, RSAD2		4
hsa-miR-3141		IFIT3, MX1, IFIT1, IFIT2		4
hsa-miR-4281		IFIT3, MX1, IFIT1, RSAD2		4
hsa-miR-6756-5p		IFIT3, MX1, IFIT1, IFIT2		4
hsa-miR-6833-5p		IFIT3, IFIT1, IFIT2, RSAD2		4
hsa-miR-6846-5p		IFIT3, MX1, IFIT1, IFIT2		4
hsa-miR-3679-5p		MX1, IFIT1, IFIT2, RSAD2		4
hsa-miR-3940-5p		MX1, IFIT1, IFIT2, RSAD2		4
hsa-miR-5698		MX1, IFIT1, IFIT2, RSAD2		4
hsa-miR-6870-5p		MX1, IFIT1, IFIT2, RSAD2		4

Abbreviations: miRNA, microRNA

**Table 4:** The up-regulated TFs based on DEGs related to the six hub genes

Transcription Factor		Genes targeted by Transcription Factor		Gene count (≥ 3)
NFE2		RSAD2, MX1, ISG15, IFIT1, IFIT3, IFIT2		6
ASCL2		RSAD2, MX1, ISG15, IFIT1, IFIT3, IFIT2		6
EGR2		RSAD2, ISG15, IFIT1, IFIT3, IFIT2		5
MAFB		RSAD2, ISG15, IFIT1, IFIT3, IFIT2		5
NR1H3		RSAD2, ISG15, IFIT1, IFIT3, IFIT2		5
ZNF467		RSAD2, ISG15, IFIT1, IFIT3, IFIT2		5
TFEC		RSAD2, MX1, IFIT1, IFIT3, IFIT2		5
KLF4		RSAD2, ISG15, IFIT3, IFIT2		4
POU2F2		MX1, ISG15, IFIT3, IFIT2		4
MYC		RSAD2, MX1, ISG15, IFIT1		4
HHEX		RSAD2, IFIT1, IFIT3, IFIT2		4
BCL6		IFIT1, IFIT3, IFIT2		3
CUX1		MX1, ISG15, IFIT2		3
FLI1		ISG15, IFIT3, IFIT2		3
TAL1		RSAD2, ISG15, IFIT1		3
EGR1		ISG15, IFIT3, IFIT2		3
ZNF404		IFIT1, IFIT3, IFIT2		3
ZNF705A		RSAD2, IFIT3, IFIT2		3
ZFP92		RSAD2, IFIT1, IFIT3		3
ZNF177		ISG15, IFIT1, IFIT3		3
ZNF488		IFIT1, IFIT3, IFIT2		3

Abbreviation: TF, transcription factors; DEGs, differentially expressed gene

## Discussion

The interaction of DCs with infectious agents (i.e., MTB) plays an important role in initiating immune response against a microbe [24]. As superior antigen presenting cells, DCs provide a critical link bridging innate and adaptive immunity; however, the outcome of interaction of MTB with DCs is not fully understood. In the present study, we systematically analyzed gene expression modules in MTB-stimulated DCs at a genome-wide scale using public datasets. Our data demonstrated that the proportion of activated DCs was significantly increased after 18 h of stimulation with MTB, reflecting that it takes longer for stimulation of DCs in comparison to macrophages. This finding could partly be explained by the complicated process of the host-pathogen interaction in DCs that involves antigen uptake and processing. Further PCA of RNA-seq data confirmed our hypothesis that DCs showed similar expression patterns after 18 h of stimulation with MTB, whereas indicated a remarkable difference from those stimulated for 2 h. However, a previous experimental study by Hellman reported that the activation of DCs occurred already within 2 h of stimulation and the expression of activation marks achieved a maximal level after 4 h [25]. This delayed response to pathogen may be attributed to the ability of MTB to paralyze the early immune response of the host, thereby resulting in bacterial multiplication in DCs [26].

Another interesting finding of our analysis was that the live tubercle bacilli produced a more prolonged immune response than heat-killed bacilli. Obviously, the live bacilli have the capability to in vivo replication in macrophages and DCs, thus secreting more antigens in compared with dead bacilli, which may be responsible for this difference. The prolonged immune response of DCs is of great importance to establish and maintain specific adaptive immunity to intracellular pathogens. Therefore, our data may partly explain why the previous subunit vaccines could not provide comparable protective effects compared with a live BCG vaccine. Along this line, several previous studies led to the important conclusion that live bacteria were required to induce long-lived specific immunity to intracellular pathogens [27, 28].

We also identified several hub genes conferring DC activation. KEGG pathway analysis showed significant enrichment of Hepatitis C, Epstein-Barr virus infection, RIG-I-like receptor signaling pathway, Human papillomavirus infection, Measles, Influenza A, and other pathways. KEGG pathway analysis was based on existing studies. As a result, the results indicate that these hub gene were associated with anti-infectious immunity. We can also speculate that genes may be associated with anti-TB immunity that further research is needed for these findings to be confirmed. GO pathway analysis show that a lot of GO terms were associated with the IFN pathway. Interestingly, the majority of proteins with increased abundance were the IFN inducible proteins, including IFIT1, IFIT2, IFIT3, ISG15, MX1, and RSAD2, indicating an important role of IFN for host resistance against MTB. Similar to our observations, several IFN-stimulated genes have been found to be upregulated in a human macrophage model of MTB infection [29, 30]. In a multicohort analysis, DEGs were analyzed between active pulmonary tuberculosis (PTB) and healthy controls (HCs) using blood transcriptional datasets. 62 DEGs including unregulated IFIT1 and IFIT3 mostly related to IFN-γ-mediated pathway, which were also in accordance with our results [31]. IFN-γ was also considered as diagnostic marker of MTB-specific cytokine responses to distinguish LTBI, active TB from health control [32]. IGRA have a better performance in predication MTB infection and it provided immunological evidence that T-cells from LTBI or active TB population would secrete IFN-γ after MTB or MTB-specific antigens re-exposure [32, 33]. Further research is required to elucidate the precise roles of IFN-γ for effective activation of DCs. Our ROC analysis indicates that these six hub genes may be potential diagnostic biomarkers in TB. IGRA, as a WHO recommended technology, is widely used to detect MTB infection [1]. Our study shows that the six hub genes IFN-γ release may be highly linked with IFN-γ release. According to previous study [34–38], IFN-γ signaling pathway is regulated by many upstream signaling molecular including the six hub genes. After screening upstream molecules of IFN-γ signaling, we would expand the sample size and evaluate these hub gene expression levels in antigen-stimulated PBMCs as potentially diagnostic markers for MTB infection and shorten the detection time.

ISG15 is a member of the ubiquitin family which is critical for the control of pathogen infections [39]. Previous investigators have concluded that macrophages overexpressing ISG15 have enhanced capabilities for secretion of pro-inflammatory cytokines, thereby boosting anti-virus activity, as well as demonstrated in the macrophages of BCG-infected mice [40]. For DCs, the upregulation of ISG15 is necessary for the induction of DC maturation [41], further improving antigen presentation and T cell stimulation against MTB. In addition, human patients with lack of ISG15 is associated with a severe Mendelian susceptibility to mycobacterial disease and patients with a nonsense mutation or a frameshift of ISG15 are deficient in IFN-γ–mediated immunity [42]. Hence, ISG15 plays an essential role as an IFN-γ-inducing secreted molecule for optimal antimycobacterial immunity. Therefore, we hypothesize that the excessive production of ISG15 in MTB-stimulated DCs may boost the activation of other immune cell types that are recruited to sites of microbial infection, thereby resulting in successful eradication of tubercle bacilli or could contribute to formation of a granuloma due to accumulation of cells at the site of infection.

We also acknowledged some obvious limitations in our present study. First, there were few transcript data from public databases about DC infected with MTB, which limited in-depth and comprehensive analyses. Second, although the expression levels of six selected hub genes and IFN-γ using Mo-DCs from healthy and TB individuals were confirmed by RT-qPCR or ELISA, we didn’t utilize other cellular biology methods to further explore the mechanism behind these findings related to DCs. Third, the data from flow cytometry or single cell sequencing may provide more information about DC response to MTB stimuli, which need more experiments to be performed.

To conclude, we comprehensively analyze transcription profiles associated with MTB stimulation in DCs and identify 6 hub genes involved in the activation of DCs during MTB infection. Our analysis also demonstrates that all these hub proteins with increased abundance are the IFN-γ inducible proteins, highlighting an important role of IFN-γ for host resistance against MTB. Further research is required to elucidate the precise roles of IFN-γ for effective activation of DCs.

### Electronic supplementary material

Below is the link to the electronic supplementary material.

Supplementary Material 1: Table S1 Top 10 Hub Genes of DEGs in DCs after infection of live MTB for 18, 48, and 72 hours and their common DEGs.

Supplementary Material 2: Table S5 Clinical characteristics of 4 healthy and 8 TB individuals in the RT-qPCR analysis.

Supplementary Material 3: Table S4 List of primers used in this study for the detection of target genes by real-time PCR.

Supplementary Material 4: Table S2 GO analysis for genes.Table S3 KEGG analysis for genes.

Supplementary Material 5: Figure S1. Principal component analysis (PCA). PCA was based on 52 samples divided into three groups, NI, live, and HI MTB-infected groups (A) or divided into four groups by four time points, 2h, 18h, 48h, and 72h (B) were performed. (NI, non-infected; HI, heat inactivation; MTB, Mycobacterium tuberculosis).

## Data Availability

The datasets presented in this study can be found in online repositories, further inquiries can be directed to the corresponding author.
