# Supplementary material for: Integrated bioinformatics analysis of dendritic cells hub genes reveal potential early tuberculosis diagnostic markers
Source: BMC Med Genomics. 2023 Sep 8;16:214. doi: 10.1186/s12920-023-01646-0 (PMC10492340; doi:10.1186/s12920-023-01646-0)
Supplement: Supplementary file 1 — Supplementary Material 1: Table S1 Top 10 Hub Genes of DEGs in DCs after infection of live MTB for 18, 48, and 72 hours and their common DEGs. [file 12920_2023_1646_MOESM1_ESM.docx]

**Table S1** Top 10 Hub Genes of DEGs in DCs after infection of live MTB for 18, 48, and 72 hours and their common DEGs

| **Rank** |  | **18 Hours** |  | **48 Hours** |  | **72 Hours** |  | **18-72 Hours** | |
| --- | --- | --- | --- | --- | --- | --- | --- | --- | --- |
|  |  | **MTB vs NI** |  | **MTB vs NI** |  | **MTB vs NI** |  | | **MTB vs NI** |
| 1 |  | IFIT1 |  | RSAD2 |  | RSAD2 |  | | RSAD2 |
| 2 |  | IFIT3 |  | IFIT1 |  | MX1 |  | | IFIT1 |
| 3 |  | MX1 |  | MX1 |  | ISG15 |  | | IFIT3 |
| 4 |  | RSAD2 |  | IFIT2 |  | IFIT1 |  | | ISG15 |
| 5 |  | OAS2 |  | ISG15 |  | IFIT3 |  | | MX1 |
| 6 |  | ISG15 |  | IFIT3 |  | OAS3 |  | | IFIT2 |
| 7 |  | STAT1 |  | OASL |  | OAS2 |  | | IFI6 |
| 8 |  | OAS3 |  | OAS2 |  | IFIT2 |  | | IRF7 |
| 9 |  | IFIT2 |  | MX2 |  | OASL |  | | EGR1 |
| 10 |  | OASL |  | OAS3 |  | OAS1 |  | | IRF4 |

Abbreviations: DEGs, differentially expressed genes; DCs, dendritic cells; MTB, Mycobacterium tuberculosis; NI, non-infected
