# Supplementary material for: Integrated bioinformatics analysis of dendritic cells hub genes reveal potential early tuberculosis diagnostic markers
Source: BMC Med Genomics. 2023 Sep 8;16:214. doi: 10.1186/s12920-023-01646-0 (PMC10492340; doi:10.1186/s12920-023-01646-0)
Supplement: Supplementary file 2 — Supplementary Material 2: Table S5 Clinical characteristics of 4 healthy and 8 TB individuals in the RT-qPCR analysis. [file 12920_2023_1646_MOESM2_ESM.docx]

**Table S5** Clinical characteristics of 4 healthy and 8 TB individuals in the RT-qPCR analysis

| Charateristics |  | Healthy Population(N=4) | TB Population（N=8） |
| --- | --- | --- | --- |
| Age, years，mean±SD |  | 26.75±2.5 | 33.12±11.32 |
| Sex, n (%) |  |  |  |
| Male |  | 2(50%) | 2(25%) |
| Female |  | 2(50%) | 6(75%) |
| BMI(Kg/m^2^), mean±SD |  | 23.12±3.67 | 19.91±2.64 |
| Ethnicity |  |  |  |
| Han-Population |  | 4(100%) | 8(100%) |
| Others |  | 0(0%) | 0(0%) |
| HIV status, n(%) |  |  |  |
| Negtive |  | 4(100%) | 8(100%) |
| Positive |  | 0(0%) | 0(0%) |

**Abbreviation**: TB, tuberculosis; RT-qPCR, real time quantitative polymerase chain reaction; SD, standard deviation; BMI, body mass index; HIV, human immunodeficiency virus
