# Supplementary material for: Integrated bioinformatics analysis of dendritic cells hub genes reveal potential early tuberculosis diagnostic markers
Source: BMC Med Genomics. 2023 Sep 8;16:214. doi: 10.1186/s12920-023-01646-0 (PMC10492340; doi:10.1186/s12920-023-01646-0)
Supplement: Supplementary file 3 — Supplementary Material 3: Table S4 List of primers used in this study for the detection of target genes by real-time PCR. [file 12920_2023_1646_MOESM3_ESM.docx]

**Table S4** List of primers used in this study for the detection of target genes by real-time PCR.

| Primer sequences 5′‐ 3′ | Target region |
| --- | --- |
| F=TTGATGACGATGAAATGCCTGA | IFIT1 gene |
| R=CAGGTCACCAGACTCCTCAC |  |
| F=AAGCACCTCAAAGGGCAAAAC | IFIT2 gene |
| R=TCGGCCCATGTGATAGTAGAC |  |
| F=TCAGAAGTCTAGTCACTTGGGG | IFIT3 gene |
| R=ACACCTTCGCCCTTTCATTTC |  |
| F=CGCAGATCACCCAGAAGATCG | ISIG5 gene |
| R=TTCGTCGCATTTGTCCACCA |  |
| F=GTTTCCGAAGTGGACATCGCA | MX1 gene |
| R=CTGCACAGGTTGTTCTCAGC |  |
| F=TGGGTGCTTACACCTGCTG | RSAD2 gene |
| R=GAAGTGATAGTTGACGCTGGTT |  |
