# Supplementary material for: Integrated bioinformatics analysis of dendritic cells hub genes reveal potential early tuberculosis diagnostic markers
Source: BMC Med Genomics. 2023 Sep 8;16:214. doi: 10.1186/s12920-023-01646-0 (PMC10492340; doi:10.1186/s12920-023-01646-0)
Supplement: Supplementary file 5 — Supplementary Material 5: Figure S1. Principal component analysis (PCA). PCA was based on 52 samples divided into three groups, NI, live, and HI MTB-infected groups (A) or divided into four groups by four time points, 2h, 18h, 48h, and 72h (B) were performed. (NI, non-infected; HI, heat inactivation; MTB, Mycobacterium tuberculosis). [file 12920_2023_1646_MOESM5_ESM.docx]

**Supplementary figure legend**


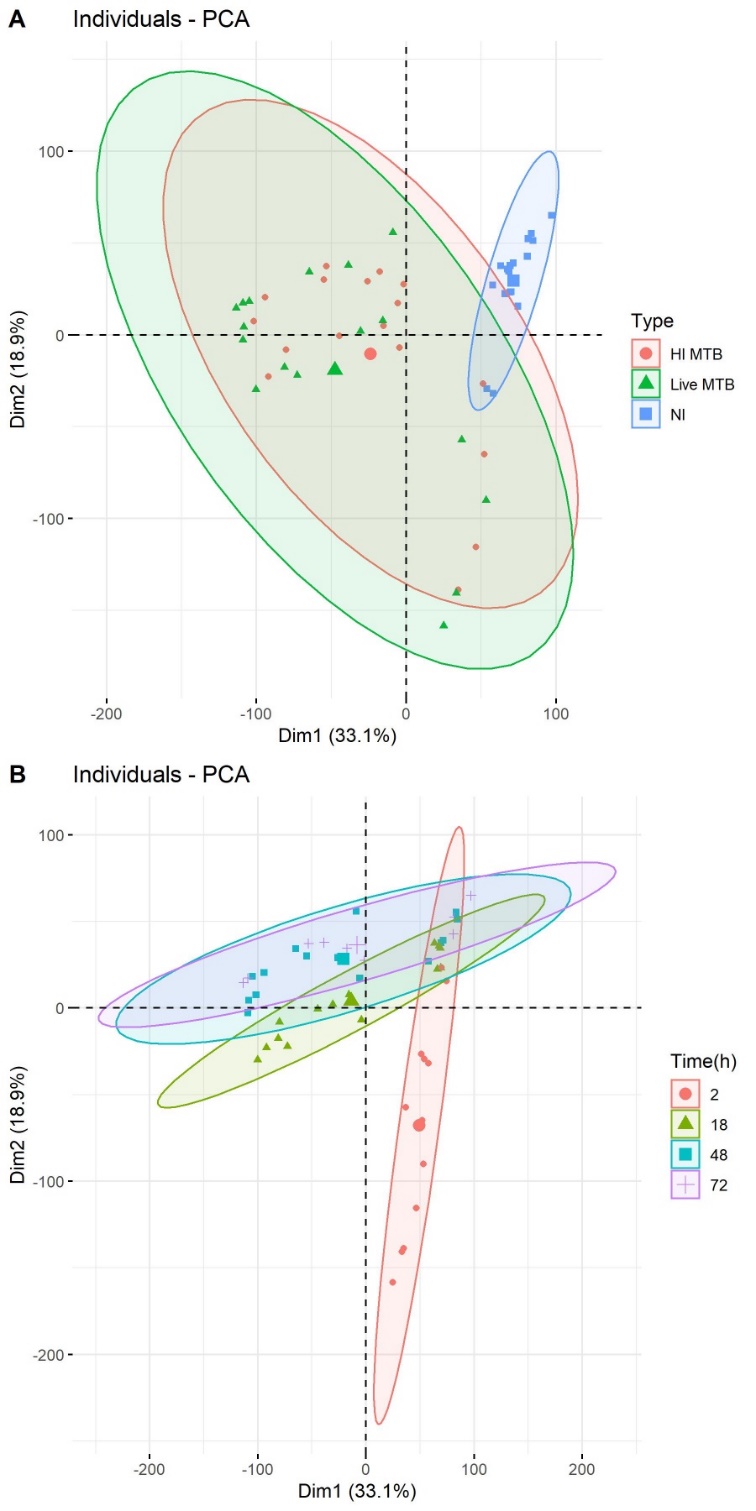


**Figure S1**. **Principal component analysis (PCA).**

PCA was based on 52 samples divided into three groups, NI, live, and HI MTB-infected groups (A) or divided into four groups by four time points, 2h, 18h, 48h, and 72h (B) were performed. (NI, non-infected; HI, heat inactivation; MTB, *Mycobacterium tuberculosis*)
